# Lysine suppresses myofibrillar protein degradation by regulating the autophagic-lysosomal system through phosphorylation of Akt in C2C12 cells

**DOI:** 10.1186/2193-1801-3-584

**Published:** 2014-10-08

**Authors:** Tomonori Sato, Yoshiaki Ito, Takashi Nagasawa

**Affiliations:** Department of Bioresources Science, The United Graduate School of Agricultural Sciences, Iwate University, Morioka, Iwate 020-8550 Japan; Department of Biological Chemistry and Food Science, Graduate School of Agriculture, Iwate University, Morioka, Iwate 020-8550 Japan

**Keywords:** Lysine, Proteolysis, C2C12, Akt, AMPK

## Abstract

The prevention of muscle wasting is important for maintaining quality of life, since loss of muscle mass can lead to a bedridden state and decreased resistance to diseases. The prevention of muscle wasting requires an increase in protein synthesis and a decrease in protein degradation in skeletal muscle. We previously showed that lysine (Lys) markedly suppressed myofibrillar protein degradation by inhibiting the autophagic-lysosomal system via the mammalian target of rapamycin (mTOR) and other signal molecules in C2C12 cells. In this study, we investigated the involvement of Akt and adenosine 5′-monophosphate (AMP)-activated protein kinase (AMPK), two regulators of autophagy, on the suppressive effects of Lys on myofibrillar protein degradation in C2C12 cells. Lys induced the phosphorylation of Akt, but the suppressive effects of Lys on myofibrillar protein degradation and autophagy were completely abolished in the presence of Akt1/2 kinase inhibitor (Akti). Lys suppressed the phosphorylation of AMPK, but this effect was also abolished by Akti. On the other hand, AMPK activation by 5-aminoimidazole-4-carboxamide-1-β-D-ribonucleoside (AICAR) did not affect either Akt activity or the autophagic-lysosomal system in C2C12 cells treated with Lys. These results indicate that regulation of AMPK activity is not essential for the regulation of autophagy by Lys. Taken together, our results show that Lys suppresses myofibrillar protein degradation by the autophagic-lysosomal system through the phosphorylation of Akt in C2C12 cells.

## Background

Skeletal muscle mass reduction is induced when the rate of protein degradation exceeds the rate of protein synthesis in skeletal muscle (Welle 1999). Such changes are caused by several catabolic diseases and by underutilization or disuse of muscle, and lead to muscle wasting (Vary and Lynch 2004; Wolfe 2006; Tisdale 2009). Muscle wasting in turn leads to decreased physical activity and ultimately to a bedridden state. Although appropriate exercise enhances protein synthesis (Mascher et al. 2007; Koopman and van Loon 2009), it is difficult for severely ill patients or the elderly to exercise sufficiently to stimulate protein synthesis. In addition, the cellular and molecular responses to exercise leading to enhanced protein synthesis are decreased in the elderly (Kim et al. 2009). Therefore, a less physically straining means7 of preventing muscle wasting is required for the elderly, infirm, and persons with disabilities. The most effective approach for these patients is to improve their nutritional state.

Leucine (Leu), one of the essential amino acids, has been reported to stimulate protein synthesis (Kimball et al. 1999; Lang et al. 2012) and to attenuate protein degradation (Nagasawa et al. 2002; Sugawara et al. 2007). We previously showed that dietary intake of Leu suppresses myofibrillar protein degradation through the autophagic-lysosomal system (Sugawara et al. 2009). Furthermore, we also demonstrated that orally administered Lys suppresses myofibrillar protein degradation in fasted rats (Sato et al. 2013), and that the suppressive effect of Lys on myofibrillar protein degradation is mediated by the autophagic-lysosomal system in C2C12 myotubes (Sato et al. 2014). In a previous study, we established that the mTOR pathway is involved in the suppressive effect of Lys on the autophagic-lysosomal system (Sato et al. 2014). However, the suppressive effect of Lys may be mediated through a signal molecule other than mTOR because the contribution of the mTOR pathway is limited.

We observed the activation of Akt in C2C12 cells treated with Lys (Sato et al. 2014). Akt is reported to regulate protein degradation through the autophagic-lysosomal system (Arico et al. 2001; Mammucari et al. 2008). AMPK is also known to regulate autophagic-lysosomal activity (Sanchez et al. 2012). In addition, it has been reported that Akt activity negatively regulates phosphorylation of AMPK (Kovacic et al. 2003). Although Akt phosphorylation is induced by Lys, the involvement of Akt phosphorylation on the regulation of myofibrillar protein degradation and on AMPK activity remains unclear.

In this study, we investigated whether Lys suppresses myofibrillar protein degradation through regulation of the Akt and/or AMPK pathway in C2C12 cells.

## Materials and methods

### Materials

Fetal bovine serum (FBS) and horse serum (HS) were purchased from BioWest (Nuaillé, France) and Invitrogen (Carlsbad, CA, USA), respectively. Dulbecco’s modified Eagle’s medium (DMEM, low glucose), minimum essential medium (MEM) Vitamin mix, Akti and dimethyl sulfoxide (DMSO) were obtained from Sigma (St. Louis, MO, USA). HEPES was obtained from Dojindo Molecular Technologies, Inc. (Kumamoto, Japan). LC3B antibody, AMPKα rabbit mAb, phospho-AMPKα (Thr172) rabbit mAb and AICAR were obtained from Cell Signaling Technology, Inc. (Danvers, MA, USA). 4E-BP1(R-113), Akt1 (B-1) and p-Akt 1/2/3 (Ser 473) were obtained from Santa Cruz Biotechnology, Inc. (Santa Cruz, CA, USA). HRP-conjugated goat anti-rabbit IgG and HRP-conjugated goat anti-mouse IgG were obtained from Kirkegaard & Perry Laboratories, Inc. (Gaithersburg, MD, USA). Other chemicals were obtained from Wako Pure Chemical Industries Ltd. (Osaka, Japan).

### Cell culture

C2C12 myoblasts (5.0 × 10^4^ cells/cm^2^) were seeded into DMEM containing antibiotics (100 units/mL penicillin and 100 μg/mL streptomycin), 10% (v/v) FBS and 44 mM sodium bicarbonate, and then cultured for 2 days. Fusion and differentiation of myoblasts into myotubes were induced by replacing the medium containing 10% FBS with medium containing 2% (v/v) HS. Fusion and differentiation conditions were maintained for 6 days, during which the medium was changed daily.

### Measurement of myofibrillar protein degradation

Since 3-methylhistidine (MeHis) is primarily in myosin and actin, two myofibrillar proteins and is not reused for protein synthesis (Young and Munro 1978), the rate of MeHis release from muscle cells directly reflects the rate of myofibrillar protein degradation (Sato et al. 2014). We measured the rates of MeHis release from C2C12 as described previously (Sato et al. 2014). In brief, myotube cells were rinsed twice with phosphate buffered saline (PBS) after 6 days of differentiation. Then, the cells were cultured for 4 h in serum and amino acid (AA)-deficient medium ((C): [1 mg ferric nitrate・9H_2_O/L, 0.4 g KCl/L, 6.4 g NaCl/L, 0.142 g NaH_2_PO_4_・2H_2_O/L, 1.0 g glucose/L, 0.265 g CaCl_2_・2H_2_O/L, 0.2 g MgSO_4_・7H_2_O/L, 0.1% (w/v) bovine serum albumin (BSA), 1% (v/v) MEM Vitamin mix, 20 mM HEPES and antibiotics]), serum and AA-deficient medium containing 10 μM Akti (Ai), serum and AA-deficient medium containing 10 mM Lys (K), serum and AA-deficient medium containing 10 mM Lys and 10 μM Akti (AiK), serum and AA-deficient medium containing 1 mM AICAR (Ac), or serum and AA-deficient medium containing 10 mM Lys and 1 mM AICAR (AcK). The rate of myofibrillar protein degradation was measured during the 4 h culture period.

### Western blot analysis

After 6 days of differentiation, the myotube cells were rinsed twice with PBS and incubated with DMEM containing 0.1% (w/v) BSA for 6 h. Then, the medium was replaced with new DMEM containing 0.1% (w/v) BSA, DMEM containing 0.1% (w/v) BSA and 10 mM Leu, DMEM containing 0.1% (w/v) BSA and 10 mM Lys, or DMEM containing 0.1% (w/v) BSA and 10 mM Gly. Akti (10 μM) or AICAR (1 mM) dissolved in DMSO (final concentration of 0.05% (v/v)) or DMSO alone (final concentration of 0.05% (v/v)) was added to the medium 30 min before amino acid stimulation. Myotubes in DMEM containing 0.1% (w/v) BSA with 10 mM Leu (L), 10 mM Lys (K), 10 mM Gly (G), or without amino acids supplementation (C) were cultured for 30 min, treated with lysis buffer solution (1% (v/v) Triton-X, 5% (w/v) deoxycholic acid, 0.1% (v/w) sodium dodecyl sulfate (SDS), 20 mM Tris–HCl (pH 7.4), 150 mM sodium chloride, 0.5 mM sodium orthovanadate (V) and 5 mM EDTA), then collected. Collected myotubes were centrifuged at 4°C, 17,900 × *g* for 10 min, then the supernatant was subjected to SDS-PAGE. Equal amounts of protein from each of the samples were separated on 10% SDS-PAGE gels, then transferred to a PVDF membrane (Millipore Corporation, Billerica, MA, USA). The membrane was blocked for 1 h with 5% skim milk in Tris buffered saline (TBS) containing 0.1% Tween 20 (TBS-T) at room temperature. The membrane was incubated overnight at 4°C with primary antibodies, then the membrane was incubated with HRP-conjugated goat anti-rabbit IgG or HRP-conjugated goat anti-mouse IgG in TBS-T. The secondary antibody was detected using an ECL western blot detection kit (GE Healthcare, Tokyo, Japan). The bands were scanned using a luminescent image analyzer (ImageQuant LAS 4000, GE Healthcare) and the relative intensity of each band was estimated using NIH Image.

### Gene expression of ubiquitin-ligase

The myotube cells were rinsed twice with PBS after 6 days of differentiation, then cultured in serum and AA-deficient medium for 1 h. Then, the cells were incubated in serum and AA-deficient medium (C) containing either 1 mM AICAR (Ac), 10 mM Lys (K), or 10 mM Lys and 1 mM AICAR (AcK), for 30 min or 4 h. Subsequently, the mRNA expressions of E3 ubiquitin ligases, muscle ring-finger protein 1 (MuRF1) and atrogin-1 were measured. Total RNA was extracted from the treated cells at the indicated time. Ten micrograms of total RNA from each C2C12 culture were separated on a 1.2% agarose-formaldehyde gel and transferred to a positively-charged nylon membrane (Pall Corporation, Port Washington, NY, USA). After UV cross-linking, membranes were hybridized with a digoxigenin-labeled cDNA probe specific to MuRF1, atrogin-1 or GAPDH for 12 to 16 h at 50°C in hybridization solution (5× SSC, 50% formamide, 50 mM sodium phosphate buffer, pH 7.0, 7% SDS, 2% blocking reagent (Roche Diagnostics, Mannheim, Germany), and 0.1% N-lauroylsarcosine). Membranes were washed twice with 2× SSC-0.1% SDS for 15 min at room temperature and twice with 0.1× SSC-0.1% SDS for 15 min at 68°C. Specific hybridization was detected with an anti-digoxigenin antibody conjugated with alkaline phosphatase, and blots were developed with CDP-star reagent (Tropix, Bedford, MA, USA). The bands were scanned using a luminescence image analyzer (ImageQuant LAS 4000) and the relative intensity of each band was estimated using NIH Image.

### Statistical analysis

Data are expressed as mean with SE. Data analysis was performed using GraphPad Instat Software version 3.0a (2001, GraphPad Software, Inc., San Diego, CA, USA). Data were analyzed by analysis of variance (ANOVA) and Tukey’s post-test in multigroup comparisons to determine whether there were significant differences (p < 0.05) among the groups.

## Results

### Activation of the Akt pathway by Lys suppresses myofibrillar protein degradation through regulation of the autophagic-lysosomal system

**Figure 1:**
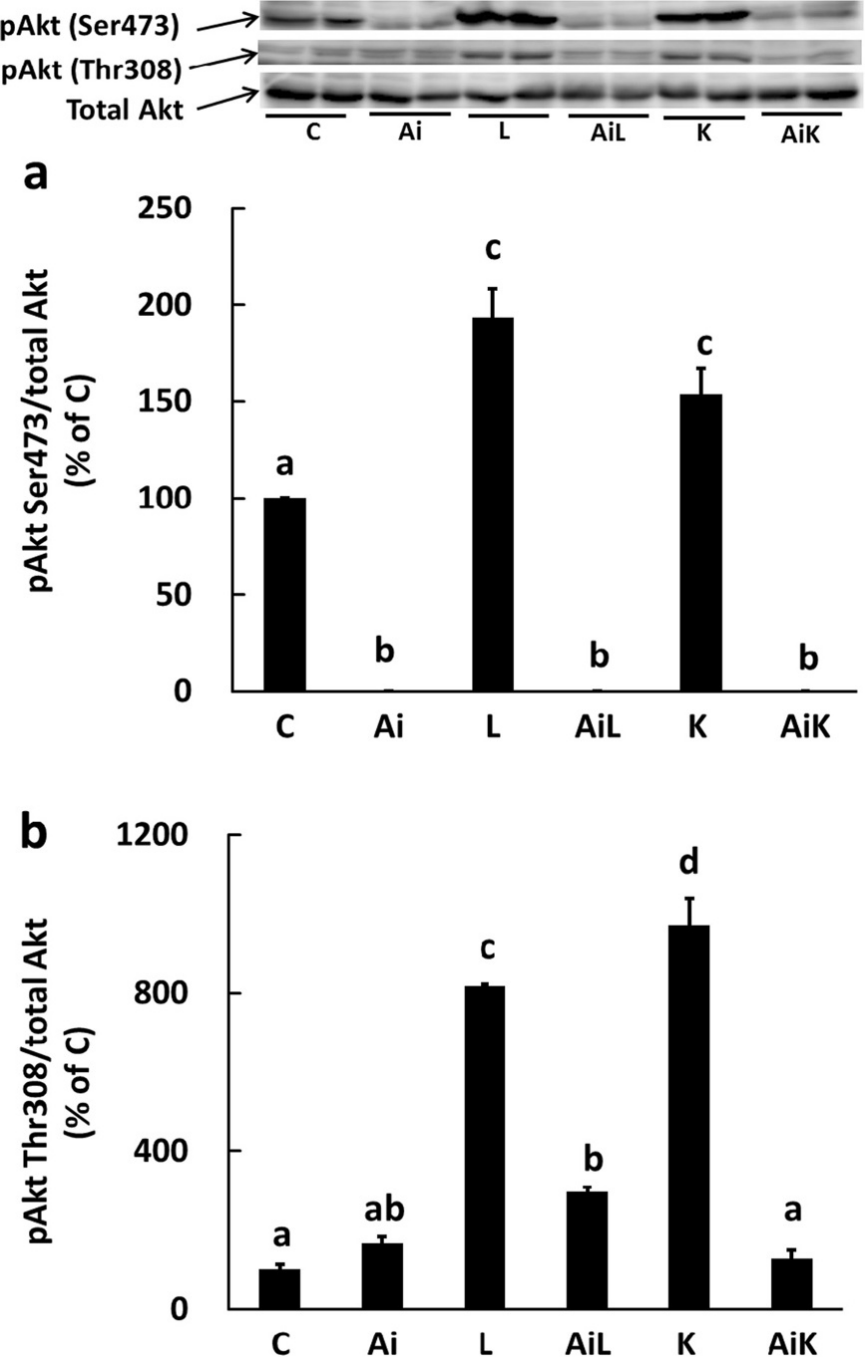
**Lys induces phosphorylation of Akt in C2C12 myotubes.** C2C12 myotubes were treated for 30 min with DMSO (C), 10 μM Akti (Ai), 10 mM Leu (L), 10 mM Leu and 10 μM Akti (AiL), 10 mM Lys (K), or 10 mM Lys and 10 μM Akti (AiK). The phosphorylation level of Akt Ser473 **(a)** and Akt Thr308 **(b)** in the lysates was analyzed by Western blotting. Results are expressed as the level relative to the expression level of the control group. Representative immunoblots are shown. Values are means with SE (n = 3–4). Different letters indicate significant differences among the groups (p < 0.05).

The phosphorylation levels of Akt Ser473 and Thr308 were significantly increased upon stimulation with 10 mM Leu (L) or 10 mM Lys (K), and these up-regulations of Akt phosphorylation were abolished by Akti (Figure 1).

**Figure 2:**
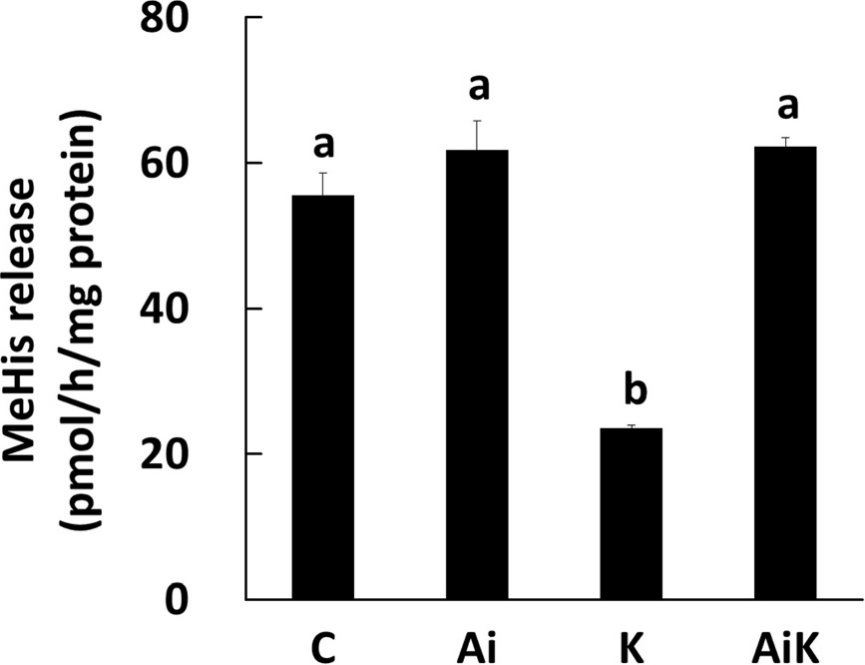
**Lys suppresses myofibrillar protein degradation through activation of Akt in C2C12 myotubes.** Cells were incubated in medium containing DMSO (C), 10 μM Akti (Ai), 10 mM Lys (K), or 10 mM Lys with 10 μM Akti (AiK) for 4 h. The amount of MeHis released from the cells was measured by HPLC. Values are means with SE (n = 3). Different letters indicate significant differences among the groups (p < 0.05).

To investigate whether the suppressive effect of Lys on myofibrillar protein degradation depends on the activation of Akt, we measured the rate of myofibrillar protein degradation in C2C12 myotubes treated with 10 mM Lys and 10 μM Akti. Lys did not suppress myofibrillar protein degradation when Akt activity was inhibited by Akti (AiK), whereas treatment with Lys alone (K) markedly suppressed myofibrillar protein degradation (Figure 2).

**Figure 3:**
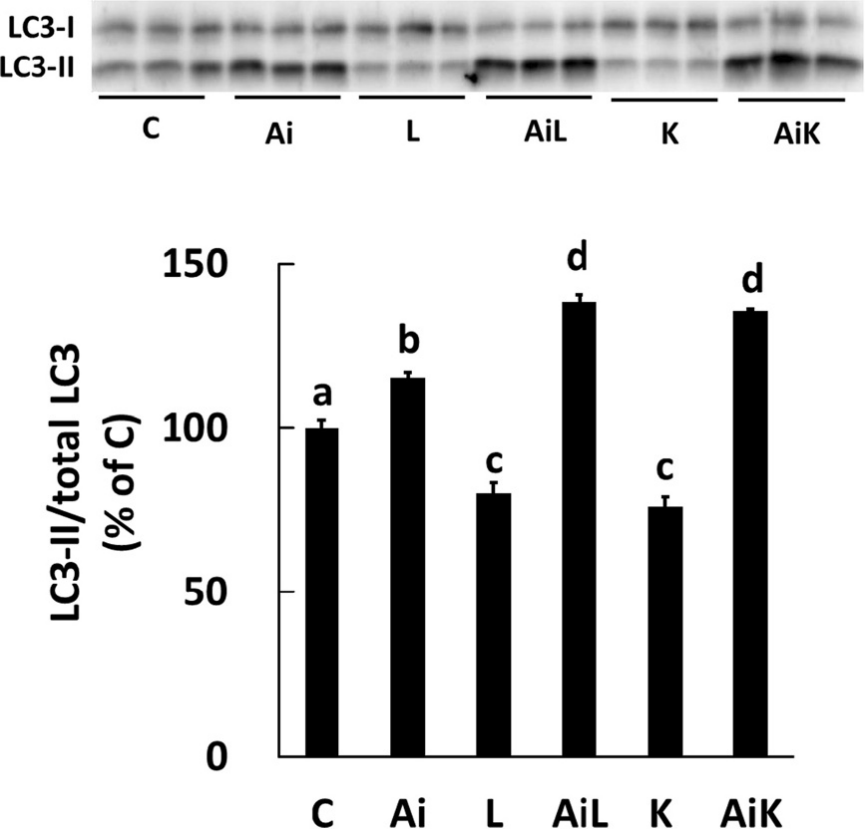
**Lys suppresses the autophagic-lysosomal system through activation of Akt in C2C12 myotubes.** Cells were treated for 30 min with DMSO (C), 10 μM Akti (Ai), 10 mM Leu (L), 10 mM Leu and 10 μM Akti (AiL), 10 mM Lys (K), or 10 mM Lys and 10 μM Akti (AiK). The ratio of LC3-II to total LC3 (LC3-I + LC3-II) in the lysates was determined by Western blotting. Results are expressed as the level relative to the control group. Values are means with SE (n = 3–4). Different letters indicate significant differences among the groups (p < 0.05).

Light chain 3 (LC3) is a mammalian homolog of Apg8p, a protein that is essential for the autophagic-lysosomal system in yeast. LC3 has two forms: LC3-I and LC3-II. The ratio of LC3-II to total LC3 (LC3-I + LC3-II) was used as a marker of autophagy (Naito et al. 2013). We evaluated the involvement of Akt activation induced by Lys in the regulation of autophagic-lysosomal activity by the ratio of LC3-II to total LC3. The ratio of LC3-II to total LC3 was reduced in C2C12 cells treated with Lys (K), however, this reduction was abolished by treatment with Akt inhibitor (AiK). Similar results were observed when the cells were treated with Leu (Figure 3). These results suggest that the suppression of the autophagic-lysosomal system by Lys is dependent on the activation of Akt.

### Lys regulates the mTOR and AMPK pathways through activation of Akt

**Figure 4:**
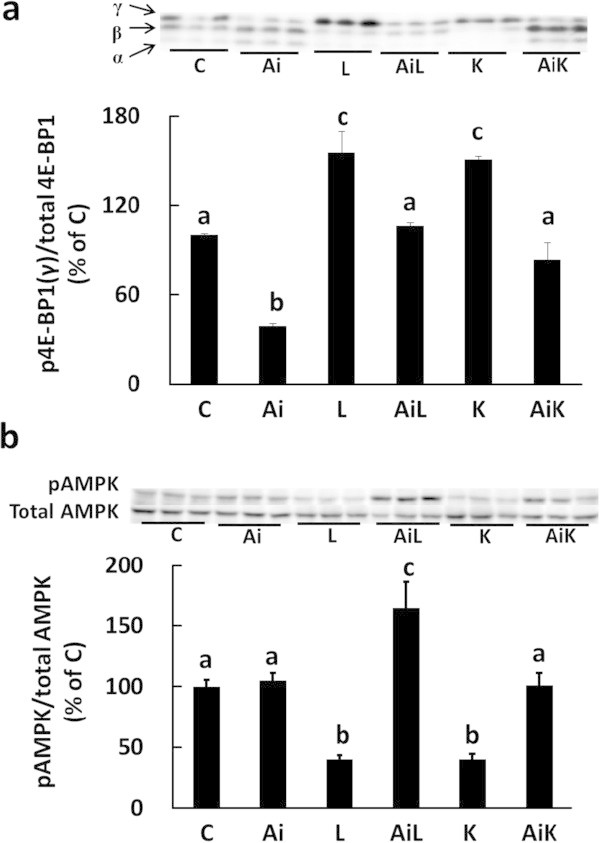
**Lys regulates the mTOR and AMPK pathways by activating Akt in C2C12 myotubes.** Cells were treated for 30 min with DMSO (C), 10 μM Akti (Ai), 10 mM Leu (L), 10 mM Leu and 10 μM Akti (AiL), 10 mM Lys (K), or 10 mM Lys and 10 μM Akti (AiK). The phosphorylation level of 4E-BP1 **(a)** and AMPK **(b)** in the lysates was analyzed by Western blotting. The level of 4E-BP1 phosphorylation was estimated from the ratio of the γ-form to that of total 4E-BP1. Results are expressed as the level relative to the level in the control group. Representative immunoblots are shown. Values are means with SE (n = 3–4). Different letters indicate significant differences among the groups (p < 0.05).

Next, we investigated the effect of Akt activation by Leu and Lys on the mTOR and AMPK pathways. One downstream target of mTOR, eIF4E-binding protein 1 (4E-BP1), was significantly phosphorylated by treatment with Leu (L) or Lys (K), and the up-regulation of 4E-BP1 phosphorylation was abolished by Akti (AiK) (Figure 4a). The level of AMPK phosphorylation was suppressed by 40% when the medium was supplemented with Leu or Lys, and the suppressive effect of Lys on AMPK phosphorylation was abolished by Akti (Figure 4b). These results indicated that Lys regulates the mTOR and AMPK pathways through Akt activation.

### Akt plays a dominant role in the regulation of autophagic-lysosomal activity compared to the AMPK pathway

**Figure 5:**
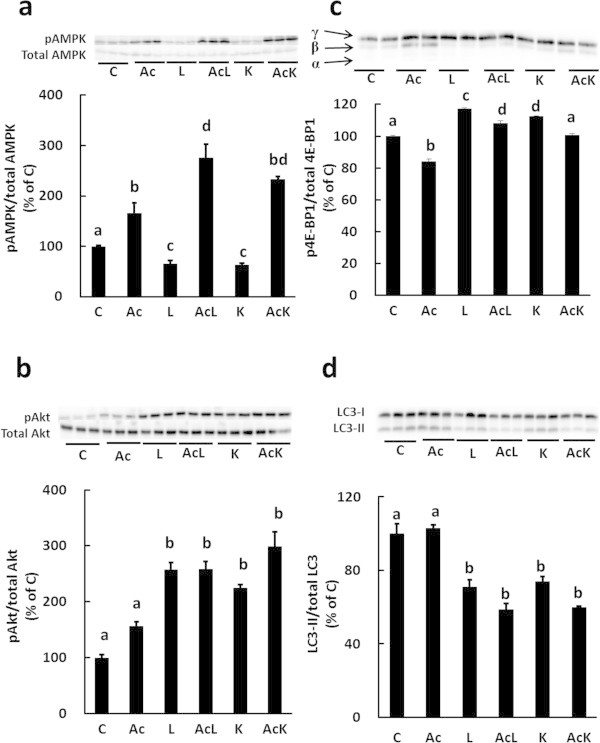
**Effect of Lys and/or AICAR treatment on activation of the AMPK, Akt and mTOR pathways, and on the autophagic-lysosomal system in C2C12 myotubes.** Cells were treated with DMSO (C), 1 mM AICAR (Ac), 10 mM Leu (L), 10 mM Leu and 1 mM AICAR (AcL), 10 mM Lys (K), or 10 mM Lys and 1 mM AICAR (AcK) for 30 min. The lysates were analyzed for phosphorylation levels of AMPK **(a)**, Akt **(b)** and 4E-BP1 **(c)**, and the ratio of LC3-II to total LC3 (LC3-I + LC3-II) **(d)**, by Western blotting. Results are expressed as the level relative to the control group. Values are means with SE (n = 3–4). Different letters indicate significant differences among the groups (p < 0.05).

We addressed the physiological role of the regulation of AMPK activity by Akt. To do this, we examined three effects of AICAR: on the phosphorylation of Akt, on autophagic-lysosomal activity, and on MeHis release upon Lys treatment. It was previously reported that the AMPK pathway induces myofibrillar protein degradation (Nakashima and Yakabe 2007). We found that AICAR treatment induced AMPK activation, and that this activation was not suppressed by Leu or Lys treatment (Figure 5a). Activation of Akt by Leu or Lys was also not affected by AICAR treatment (Figure 5b). The ratio of LC3-II to total LC3 was unaffected by AICAR treatment, but the ratio decreased when Leu or Lys was added to the medium, even in the presence of AICAR (Figure 5d). These results suggested that Akt, rather than AMPK pathway, might dominantly regulate autophagic-lysosomal activity.

The mTOR pathway is suppressed by the AMPK pathway (Bolster et al. 2002), and the mTOR pathway is involved in the suppressive effect of Lys on autophagic-lysosomal activity (Sato et al. 2014). Consequently, we investigated whether mTOR activity is affected by AMPK activation. Activation of the AMPK pathway by AICAR decreased the basal phosphorylation level of 4E-BP1 and reduced the phosphorylation levels of 4E-BP1 induced by Leu or Lys (Figure 5c). These results suggest that mTOR activity may not be critical for the regulation of autophagic-lysosomal activity by Lys.

**Figure 6:**
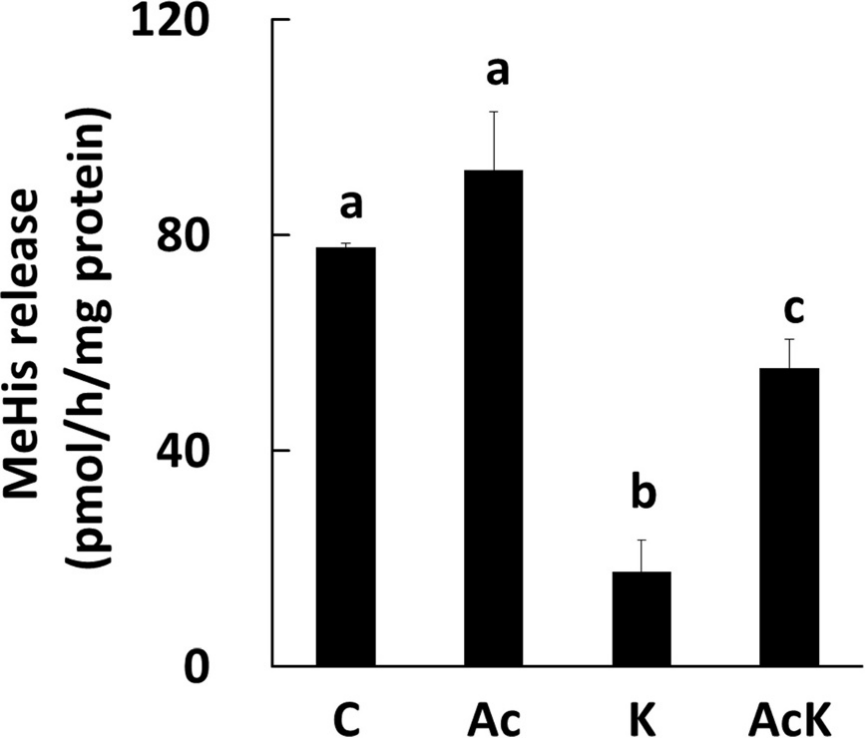
**Effect of Lys and/or AICAR treatment on myofibrillar protein degradation in C2C12 myotubes.** Cells were incubated in medium containing DMSO (C), 1 mM AICAR (Ac), 10 mM Lys (K), or 10 mM Lys and 1 mM AICAR (AcK) for 4 h. MeHis released from the cells was measured by HPLC. Values are means with SE (n = 3). Different letters indicate significant differences among the groups (p < 0.05).

**Figure 7:**
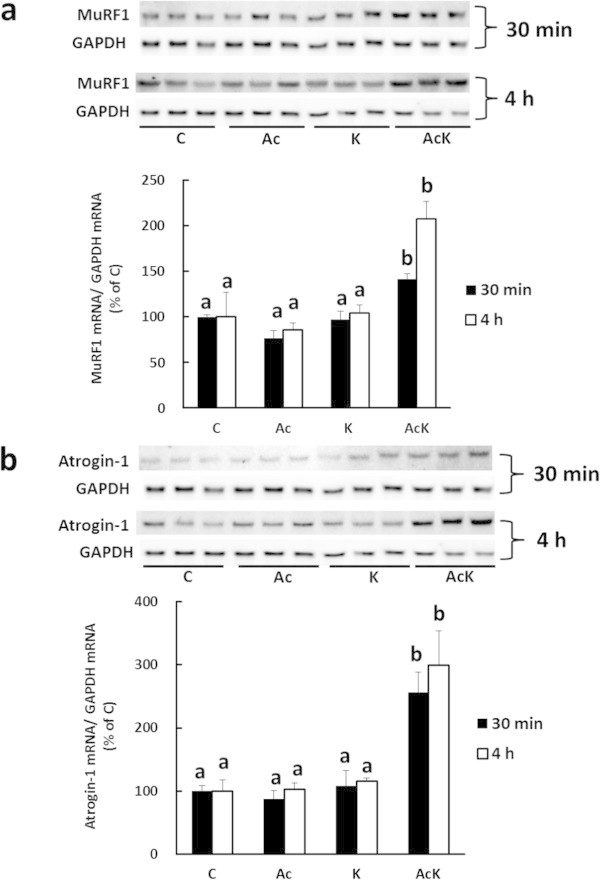
**Effect of Lys and/or AICAR on gene expression of MuRF1 and atrogin-1 mRNA in C2C12 myotubes.** Cells were treated with DMSO (C), 1 mM AICAR (Ac), 10 mM Lys (K), or 10 mM Lys and 1 mM AICAR (AcK) for 30 min (filled bar) or 4 h (open bar). The expression of MuRF1 **(a)** and atrogin-1 **(b)** mRNA was analyzed as described in “Materials and Methods”. Results are shown as expression relative to the control group. Values are means with SE (n = 3). Different letters indicate significant differences among the groups at the same time point (p < 0.05).

MeHis release is an index of myofibrillar protein degradation. MeHis release increased in the presence of AICAR and Lys compared to in the presence of Lys alone (Figure 6). On the other hand, autophagic-lysosomal activity (the ratio of LC3-II to total LC3) was sufficiently suppressed by Lys in the presence of AICAR (Figure 5d). AICAR treatment was reported to induce the expression of the E3 ubiquitin ligase genes, MuRF1 and atrogin-1, in C2C12 myotubes (Nakashima and Yakabe 2007); therefore, we examined whether AICAR treatment stimulates the ubiquitin-proteasomal system. The expression of MuRF1 and atrogin-1 mRNA significantly increased in C2C12 cells treated with Lys and AICAR (AcK), whereas treatment with either AICAR or Lys alone (Ac, K) did not affect MuRF1 and atrogin-1 mRNA levels (Figure 7). Therefore, the increase in MeHis release may reflect activation of the ubiquitin-proteasomal system in cells treated with Lys and AICAR.

### Glycine slightly induces Akt activation and does not regulate the phosphorylation of AMPK and the autophagic-lysosomal system

**Figure 8:**
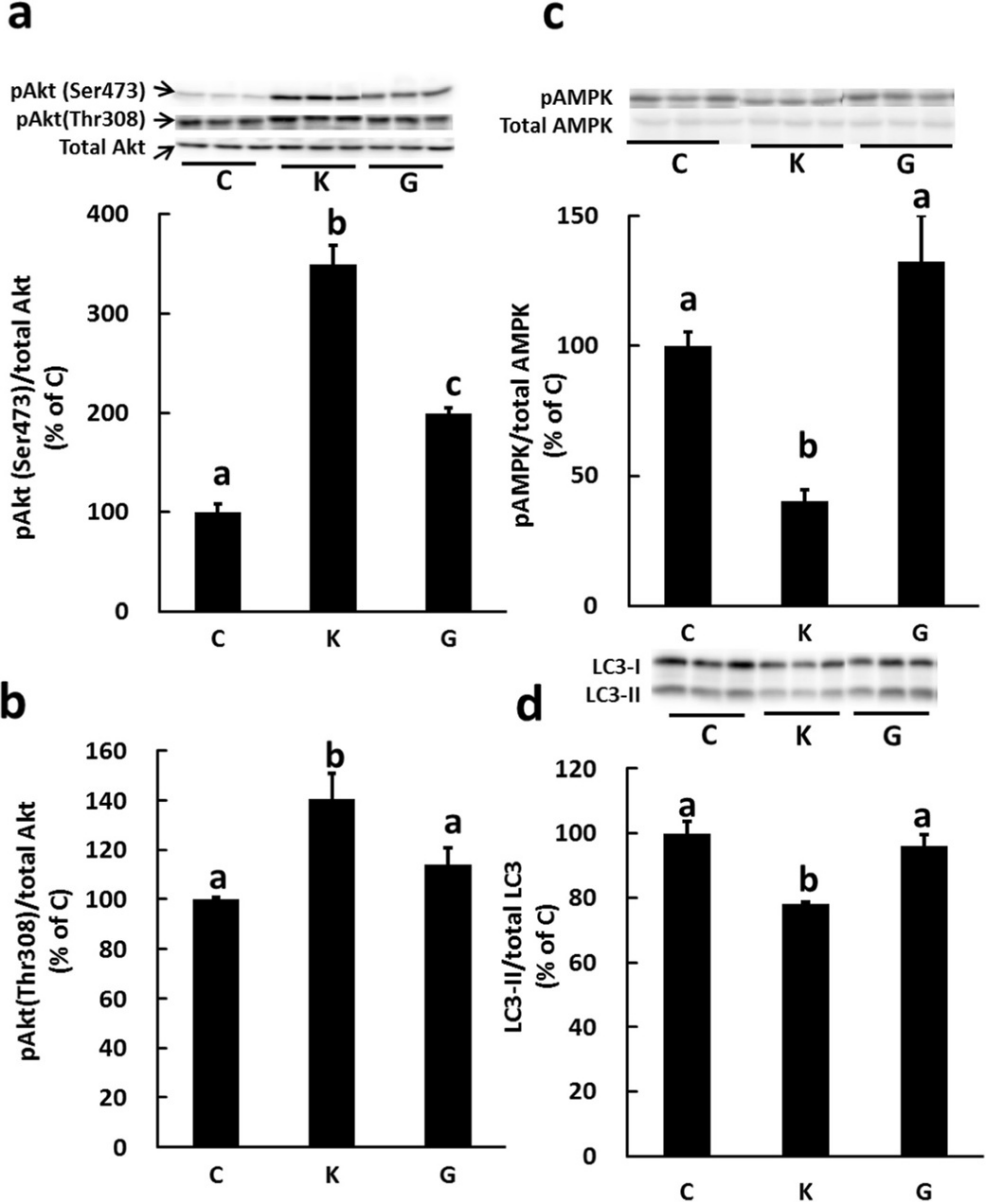
**Comparison of Lys and Gly stimulation on the phosphorylation of Akt and AMPK, and on the activity of the autophagic-lysosomal system in C2C12 myotubes.** Cells were untreated (C) or treated with 10 mM Lys (K) or 10 mM Gly (G) for 30 min. The phosphorylation levels of Ser473 in Akt **(a)**, Thr308 in Akt **(b)**, AMPK **(c)**, and the ratio of LC3-II to total LC3 (LC3-I + LC3-II) **(d)**, were determined in the lysates by Western blotting. Results are expressed as the level relative to that of the control group. Values are means with SE (n = 3–4). Different letters indicate significant differences among the groups (p < 0.05).

We previously showed that Gly does not suppress myofibrillar protein degradation in C2C12 myotubes (Sato et al. 2014). However, it is still unknown whether Gly regulates the activities of autophagy and signal molecules which regulate autophagy. We therefore investigated the effect of Gly on the phosphorylation levels of Akt and AMPK, and on the activity of the autophagic-lysosomal system in C2C12 myotubes. The phosphorylation level of Akt Ser473 slightly increased upon Gly treatment, but the level was much lower than that obtained following Lys stimulation. In addition, the phosphorylation level of Akt Thr308 did not increase upon Gly treatment (Figure 8a, b). Furthermore, Gly did not affect the phosphorylation of AMPK (Figure 8c), and the ratio of LC3-II to total LC3 was not changed by Gly treatment (Figure 8d). These results demonstrate that regulation of the Akt and AMPK pathways, and of the autophagic-lysosomal system, is specific to Lys, and that the effect of Lys is not merely due to it being a supplemental nitrogen source.

## Discussion

Leu is known to activate mTOR signaling, which stimulates muscle protein synthesis and suppresses protein degradation (Kimball et al. 1999; Kim et al. 2009). We previously showed that Lys suppresses myofibrillar protein degradation through mTOR signaling and another signaling pathway, and we observed significant up-regulation of Akt phosphorylation by Lys (Sato et al. 2014). Akt has been reported to regulate the autophagic-lysosomal system (Arico et al. 2001; Mammucari et al. 2008). Therefore, we here investigated whether Lys suppresses myofibrillar protein degradation by regulating the phosphorylation of Akt in C2C12 myotubes.

The phosphorylation level of Akt Ser473 significantly increased with Lys treatment (Figure 1a), as shown previously (Sato et al. 2014), and the phosphorylation level of Akt Thr308 also significantly increased in the presence of Lys (Figure 1b). The suppressive effect of Lys on myofibrillar protein degradation was completely abolished when Akt phosphorylation was inhibited by Akti (Figures 1 and 2). These results clearly demonstrate that Lys suppresses myofibrillar protein degradation by activating Akt. Similarly, the autophagic-lysosomal system in C2C12 cells treated with Akti was not suppressed by Lys, since the ratio of LC3-II to total LC3 did not decrease (Figure 3). This result indicates that activation of Akt is essential for the suppression of the autophagic-lysosomal system by Lys.

Several studies have shown that Akt is activated by the addition of amino acids. Tato et al. (2011) showed that Akt is phosphorylated following the addition of a mixture of amino acids, and Leu has been reported to activate Akt phosphorylation (Lee et al. 2008; Coëffier et al. 2011; Zeanandin et al. 2012). These studies support our finding that Lys is another stimulating amino acid that can activate the Akt pathway (Figure 1). However, the effect on Akt activation seems to be specific to certain amino acids, as Gly only slightly stimulated Akt phosphorylation and did not suppress autophagic-lysosomal activity (Figure 8). Therefore, Lys may be an important regulatory amino acid in muscle protein metabolism.

It has been reported that Akt suppresses autophagy via mTOR signaling (Janku et al. 2011; Jamart et al. 2012; Seldin et al. 2013). In this study, we found that activation of Akt by Lys regulates mTOR signaling (Figure 4a). However, we previously showed that mTOR signaling plays a limited role in the suppression of myofibrillar protein degradation by Lys (Sato et al. 2014), and it has been shown that Leu also regulates the autophagic-lysosomal system in an mTOR-independent manner (Mordier et al. 2000). Furthermore, Zhao et al. (2007) have shown that Akt, rather than mTOR, is a master regulator of autophagy in myotubes. Therefore, it is likely that the activation of Akt plays a primary role in the regulation of autophagic-lysosomal activity by Lys. In this study, we observed that Leu also regulates some signals in similar manner to Lys. From these results, the regulatory mechanism on autophagy by Lys may be similar to that by Leu. However, the stimulatory effect of Leu on mTOR signaling was significantly higher than that of Lys in this study (Figure 5c) and previous study (Sato et al. 2014). Therefore, the regulation of mTOR signaling or protein synthesis by Lys may be weaker than that by Leu. Further study is needed to clarify that similarity and difference between Leu and Lys signals on regulation of protein metabolism.

AMPK is also a regulator of autophagy (Sanchez et al. 2012). Leu has been reported to regulate protein turnover by suppressing AMPK (Du et al. 2007; Wilson et al. 2011). In this study, we showed that Lys suppresses AMPK phosphorylation, and that the suppressive effect of Lys on AMPK phosphorylation was completely abolished when Akt activity was inhibited (Figure 4b). Leu similarly regulated AMPK phosphorylation by Akt in C2C12 cells. AMPK is a downstream target of Akt (Kovacic et al. 2003; Horman et al. 2006; Bertrand et al. 2008; Suzuki et al. 2013). Therefore, the current results indicate that both Lys and Leu suppress AMPK phosphorylation through Akt activation in C2C12 cells.

C2C12 myotubes were treated with AICAR for 4 h to examine the involvement of AMPK activity in the degradation of myofibrillar protein. Treatment with AICAR alone had essentially no effect on MeHis release from cells (Figure 6). In contrast, Nakashima and Yakabe (2007) showed that activation of AMPK by AICAR stimulates myofibrillar protein degradation in C2C12 cells. This discrepancy between the two sets of results may be due to the different treatment times (24 h in the study by Nakashima and Yakabe, and 4 h in this study); alternatively, AMPK activity might have been sufficiently high in the current study to promote the degradation of myofibrillar protein even under basal conditions without AICAR stimulation. When cells were treated with Lys in the presence of AICAR, the ratio of LC3-II to total LC3 remained suppressed, and Lys did not affect the activation of AMPK by AICAR (Figure 5a and d). These results suggested that autophagic-lysosomal activity might be dominantly regulated by Akt rather than AMPK pathway in this study. Furthermore, the rate of myofibrillar protein degradation significantly increased in C2C12 cells treated with Lys and AICAR (AcK) compared to cells treated with Lys alone (K) (Figure 6). To investigate why myofibrillar protein degradation increased upon treatment with Lys and AICAR, we assessed the activity of the major proteolytic system, the ubiquitin-proteasomal system. The mRNA levels of MuRF1 and atrogin-1 were significantly increased in C2C12 cells 30 min and 4 h following the addition of Lys and AICAR (AcK) compared to the other groups (C, Ac, K) (Figure 7). Therefore, the activation of AMPK in the presence of some amino acids may stimulate the ubiquitin-proteasomal system and increase the rate of myofibrillar protein degradation in C2C12 cells, although the mechanism of this phenomenon is unclear.

On the other hand, AMPK has been shown to regulate protein synthesis through the mTOR pathway in C2C12 cells (Williamson et al. 2006) and in rats (Bolster et al. 2002). When AMPK was phosphorylated by AICAR, mTOR signaling activity was significantly suppressed compared to in the presence of Lys alone (Figure 5c). This result indicates that the suppressive effect of Lys on AMPK phosphorylation may help up-regulate protein synthesis by activating the mTOR pathway, rather than by regulating autophagic activity.

The nutritional value of Lys has been well-appreciated because Lys is a major limiting essential amino acid in some plant proteins, such as wheat gluten. Addition of Lys to low-Lys diet has been reported to help animal growth whereas animals which fed a low-Lys diet grow more slowly than those fed a diet containing adequate amounts of Lys (Nagao et al. 2010; Ishida et al. 2011). However, there is little information of molecular mechanisms in the health-promoting benefits of Lys until now. In this study, we indicated that Lys may act as regulator of autophagy and some signal molecules which control protein turnover in C2C12 cells. The current results may provide basis for the development of nutritional treatment for muscle wasting and muscle mass gain by daily diet, and may contribute to the understanding the physiological function of amino acids and its molecular mechanism in skeletal muscle cells.

In conclusion, this study demonstrated for the first time that Lys suppresses myofibrillar protein degradation by regulating the autophagic-lysosomal system through phosphorylation of Akt. Consequently, Lys is an important regulatory amino acid in protein metabolism and exerts its effect via the Akt pathway.

## Abbreviations

Akt: Protein kinase B
Akti: Akt1/2 kinase inhibitor
AICAR: 5-aminoimidazole-4-carboxamide-1-β-D-ribonucleoside
AMPK: Adenosine 5′-monophosphate (AMP)-activated protein kinase
BSA: Bovine serum albumin
DMEM: Dulbecco’s modified Eagle’s medium
DMSO: Dimethyl sulfoxide
FBS: Fetal bovine serum
Gly: Glycine
HS: Horse serum
LC3: Light chain 3
Leu: Leucine
Lys: Lysine
MuRF1: Muscle ring-finger protein 1
mTOR: Mammalian target of rapamycin
PBS: Phosphate buffered saline
MeHis: 3-methylhistidine
4E-BP1: eIF4E-binding protein 1.
